# Toward Better Utilization of the 2015 Japan Standard Population

**DOI:** 10.2188/jea.JE20230135

**Published:** 2023-10-05

**Authors:** Kota Katanoda, Hirokazu Tanaka, Sayo Tanaka, Kayo Togawa

**Affiliations:** Division of Population Data Science, National Cancer Center Institute for Cancer Control, Tokyo, Japan

**Keywords:** standard population, age-standardized mortality, the 2015 Japan Standard Population, the 1985 Japan Standard Population, direct standardization method

In a recent paper on statistical analysis, we provided the annual age-standardized mortality rates (ASMRs) for 1950–2020 using the new 2015 Japan Standard Population.^1^ In addition, we analyzed the correlation with the former ASMRs (based on 1985 Japan Standard Population) to determine the impact of the introduction of the new standard population.^1^ Although we were able to successfully calculate the new national-level ASMRs in that paper, regional data (eg, prefecture-level ASMRs) are still unavailable due to a lack of prefecture-level population data for the older population (ie, “85–89 years,” “90–94 years,” and “95 years and older”). This letter discusses practical and administrative issues associated with updating the standard population.

The first issue with the update of the standard population is that the official long-term ASMRs using the 2015 Japan Standard Population are unavailable on an annual basis. Although the Ministry of Health, Labour and Welfare (MHLW) retrospectively published the ASMR for major causes of deaths using the new standard population, the data are only every 5 years (ie, only for census years).^2^ That was the major motivation for us to provide the annual population and ASMR data in our previous paper.^1^ When it comes to regional data, however, since prefecture-level population data for the older population groups are not yet available as official statistics, except those for census years, it is still difficult to calculate and compare annual changes in ASMRs by prefecture using the new standard population. This situation may lead to the mixing of former and new ASMRs in setting targets for national health programs, such as “Health Japan 21”,^3^ and may indeed prevent utilization of the new ASMRs in practice.

In introducing the new standard population, the MHLW has convened an expert committee, the Expert Study Group for Revision of the Standard Population, three times since October 2019.^4^ These experts have discussed the necessity and impact of changing the standard population, though not so thoroughly as our previous paper.^1^ In addition, opinions have been sought from related academic societies, including the Japan Epidemiological Association and the Population Association of Japan, and the issue of lacking population data mentioned above has already been pointed out by many experts.^4^ To date, however, there has been no administrative action to address this issue, except for the provision of the national data every 5 years.

The introduction of the new standard population did have a scientific rationale, in that it subdivided the older population and reflected the mortality and morbidity trends of the older age groups more precisely in age-standardized rates. Nevertheless, the introduction process did not include adequate preparation of population data. This will, in turn, hamper use of the new age-standardized rates in long-term secular changes and regional comparisons, such as among prefectures. If policy changes were to be made, it is necessary to prepare corresponding data so that the national and regional health programs could be made and managed accordingly. The government and experts should reflect on these points and work to prepare the necessary data to disseminate and promote the use of the new age-standardized rates.
